# Characterizing Sphingosine Kinases and Sphingosine 1-Phosphate Receptors in the Mammalian Eye and Retina

**DOI:** 10.3390/ijms19123885

**Published:** 2018-12-05

**Authors:** Hunter Porter, Hui Qi, Nicole Prabhu, Richard Grambergs, Joel McRae, Blake Hopiavuori, Nawajes Mandal

**Affiliations:** 1 Department of Ophthalmology, University of Oklahoma Health Sciences Center, Oklahoma City, OK 73104, USA; hunter-porter@OUHSC.edu (H.P.); hui-qi@OUHSC.edu (H.Q.); nicole-prabhu@OUHSC.edu (N.P.); joel-mcrae@OUHSC.edu (J.M.); blake-hopiavuori@OUHSC.edu (B.H.); 2 Departments of Ophthalmology, Anatomy and Neurobiology, University of Tennessee Health Sciences Center, Memphis, TN 38163, USA; rgramber@uthsc.edu

**Keywords:** S1P, sphingosine 1-phosphate, S1PR1, sphingosine 1-phosphate receptor 1, S1PR2, S1PR3, sphingosine kinase 1, SPHK2, retina, eye

## Abstract

Sphingosine 1-phosphate (S1P) signaling regulates numerous biological processes including neurogenesis, inflammation and neovascularization. However, little is known about the role of S1P signaling in the eye. In this study, we characterize two sphingosine kinases (SPHK1 and SPHK2), which phosphorylate sphingosine to S1P, and three S1P receptors (S1PR1, S1PR2 and S1PR3) in mouse and rat eyes. We evaluated sphingosine kinase and S1P receptor gene expression at the mRNA level in various rat tissues and rat retinas exposed to light-damage, whole mouse eyes, specific eye structures, and in developing retinas. Furthermore, we determined the localization of sphingosine kinases and S1P receptors in whole rat eyes by immunohistochemistry. Our results unveiled unique expression profiles for both sphingosine kinases and each receptor in ocular tissues. Furthermore, these kinases and S1P receptors are expressed in mammalian retinal cells and the expression of SPHK1, S1PR2 and S1PR3 increased immediately after light damage, which suggests a function in apoptosis and/or light stress responses in the eye. These findings have numerous implications for understanding the role of S1P signaling in the mechanisms of ocular diseases such as retinal inflammatory and degenerative diseases, neovascular eye diseases, glaucoma and corneal diseases.

## 1. Introduction

Sphingolipids play diverse roles in the biology of cells and tissues [1]. As structural lipids, they are essential to maintaining membrane fluidity and organizing lipid rafts [2,3]. However, their role is far from limited to membrane dynamics. The various species of sphingolipids are found throughout the cell including in the nucleus, cytoplasm, and in the extracellular space as signaling molecules [3,4,5,6]. Bioactive sphingolipids such as sphingosine 1-phosphate (S1P) and ceramide (Cer) are now recognized as important mediators of many basic cellular processes including cell migration, survival, contraction, proliferation, gene expression and various cell-cell interactions [1,5,7,8,9,10,11,12,13,14,15]. The scope of the sphingolipid system’s impact on mammalian biology has proven to be impressive due to the complexity of the “sphingolipid rheostat” and the many signaling pathways that sphingolipids are involved in.

The sphingolipid rheostat is comprised of dozens of enzymes that constantly shift concentrations of sphingolipids such as sphingomyelin (SM), Cer and sphingosine (Sph) by metabolizing them into other sphingolipid species. Ceramides and S1P are potent signaling molecules, which the sphingolipid rheostat can generate to respond to a stimulus or use as intermediates toward the degradation pathway. S1P is generated from sphingosine by sphingosine kinases 1 and 2 (SPHK1/SPHK2). Mice deficient in both SPHK1 and SPHK2 have many vascular and neuronal deficits during the development and are not viable past E13.5 [16]. SPHK1 and SPHK2 single knockout animals generally have no phenotype and are thought to compensate with the remaining kinase. However, knocking out SPHK2 is sufficient to reduce resilience to oxygen-induced retinopathy [17]. Meanwhile, the S1P produced by the two kinases has shown myriad roles in retinal development and function. S1P has shown to be essential for photoreceptor development [18] and Muller glia migration [19]. After development, modulating S1P has shown benefits in preventing immune cell migration and neovascularization after insult.

The biological effects of S1P are largely attributed to its high-affinity association with receptors present on various cell types. S1P receptors are divided into five subgroups (S1PR1-5), which are G-protein-coupled receptors with unique expression patterns and roles in cell signaling [20,21,22,23,24]. S1PR1 is the most well studied and is present on endothelial cells, B-cells and T-cells. S1PR1 is also an essential receptor for influencing blood vessel development and maturation [21,25,26]. S1PR1 knockout animals are nonviable. S1PR2 and 3 are also ubiquitously expressed [20,21,22,23,24] and important for proper vascular development [27,28]. S1PR2 is important for cell migration [29] and S1PR2 mutations induce hearing loss by disrupting endocochlear potential gradients [30]. S1PR3 has demonstrated an important role in a bacterial infection response [31], nociception and itching sensation [32]. S1PR4 and S1PR5 expression is more restricted and they are mostly found on the cells of the immune and nervous systems, respectively [33,34]. S1PR4 is essential to differentiation of dendritic cells [35] and is important to immune cell chemoattraction [36]. S1PR5 is important in cell migration and, by selectively modulating its activity, can ameliorate demyelination [37] and alter the integrity of the blood brain barrier [38]. In summary, S1P receptors are essential for immune cell response modulation, cell migration, and vascular development and maintenance. These functions are critical to retinal development and survival, but their role in the eye is poorly understood.

In the eye, S1P can protect neural cells from apoptosis by inhibiting synthesis of its precursor ceramide, which is a pro-death signaling molecule [5,39,40,41,42,43]. We have previously shown that targeting S1P receptors with FTY-720, which is marketed as a drug for multiple sclerosis, can delay retinal degeneration in light damage and genetic models [44,45]. Ocular tissues outside the retina such as the cornea and retinal pigment epithelium (RPE) have shown S1P receptor expression with functions important to controlling fibrotic responses [46]. Modulating the S1P receptor signaling with FTY-720 has also shown to be beneficial in ocular autoimmune conditions such as uveitis [47]. In the retina proper, S1P is critical to photoreceptor development [18], axonal guidance [48] and survival of retinal ganglion cells in glaucoma [49]. Given the numerous effects of S1P receptors showing translation into ocular development and pathology, we have identified a critical need to understand the distributions of both S1P receptors and sphingosine kinases in the eye. As S1P receptor modulation has shown benefits in counteracting retinal degeneration, we also studied how the expression of S1P-producing sphingosine kinases and S1P receptors changes over time in cases of retinal damage induced by intense light stress. Characterizing S1P signaling in ocular development and stress responses will deepen our understanding of eye health and diseases and potentially identify novel pathways for therapeutic intervention.

## 2. Results

### 2.1. Distribution and Expression of Sphingosine Kinase (Sphk) and Sphingosine 1-Phosphate Receptor (S1pr) mRNA in Rat Tissue

**Figure 1:**
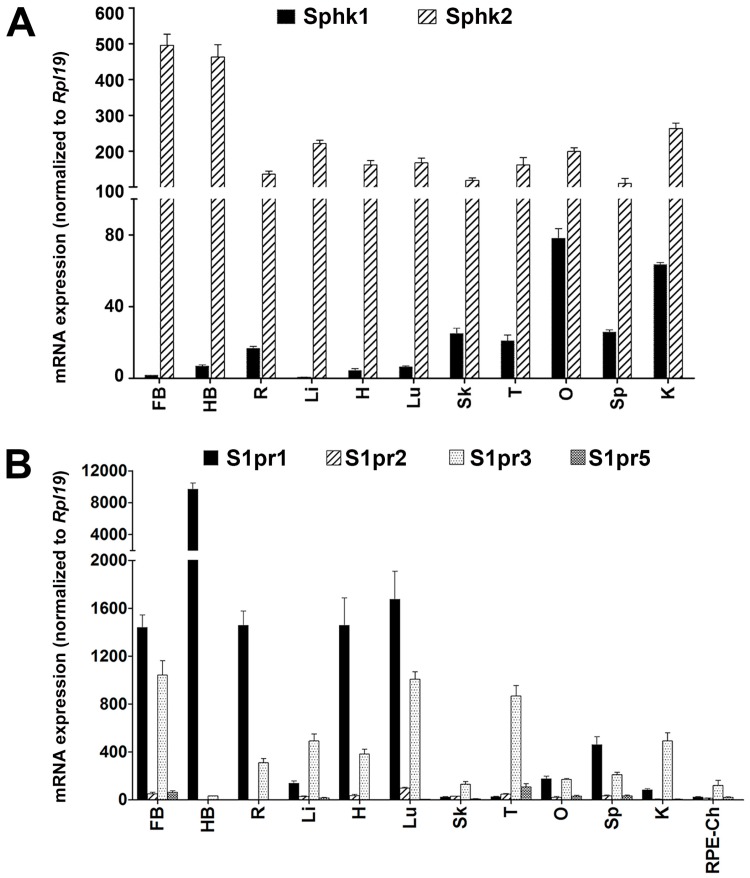
The expression and distribution of sphingosine kinases and S1P receptors in adult (2–7 months old) SD rat tissues. For each feature, the mean of three independent qRT-PCR experiments (±SE) is shown and normalized to the expression of the ribosomal protein Rpl19. (**A**) Sphingosine kinase (*Sphk*) 1 and 2 mRNA expression in the tissue. (**B**) Sphingosine 1-phosphate receptor (*S1pr*) 1, 2, 3 and 5 mRNA expression in tissue. FB, forebrain. HB, hindbrain. R, retina. Li, liver. H, heart. Lu, lung. Sk, skin. T, testes. O, ovary. Sp, spleen. K, kidney. RPE-Ch, retinal pigment epithelium-choroids.

To understand the roles of S1P signaling in the eye, we first explored the expression of relevant protein coding transcripts throughout the body. Using qRT-PCR, we assayed the expression and distribution of S1P-producing enzymes and the major S1P receptors in various tissues from adult SD rats. SPHK2 is corroborated as a major S1P-producing enzyme with high expression across all assayed tissues. At the same time, SPHK1 was expressed primarily in ovary and kidney tissue with minimal expression in most other tissues—notably the brain and retina (Figure 1A). *S1pr1* was highly expressed in the hindbrain, moderately expressed in the forebrain, retina, heart, lungs and spleen, and minimally expressed in the liver, skin and testes. *S1pr3* was moderately expressed in the forebrain, retina, liver, heart, lungs, testes and kidney and in retinal pigment epithelium-choroid (RPE-choroid) tissue. *S1pr2* and *S1pr5* showed minimal expression in the tissues analyzed (Figure 1B).

### 2.2. Distribution and Expression of Sphingosine Kinase and Major S1P Receptor mRNA in Mouse Eye Tissues

**Figure 2:**
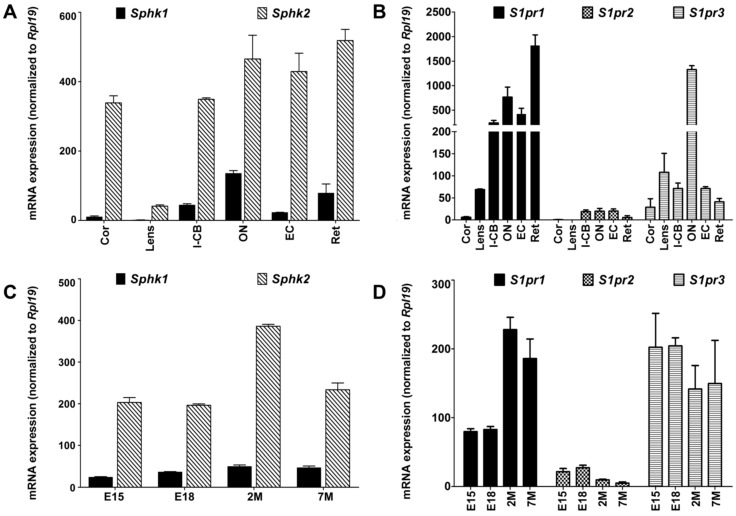

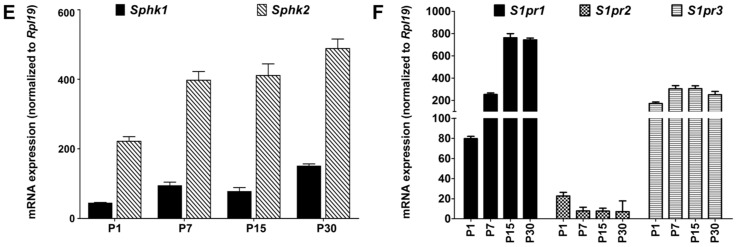
The expression and distribution of sphingosine kinase (*Sphk*) 1 and 2 and sphingosine 1-phosphate receptor (*S1pr*) 1, 2 and 3 mRNA in mouse eye tissues. Each bar shows the mean of three independent qRT-PCR experiments (±SE) normalized with the expression of the ribosomal protein Rpl19. (**A**) *Sphk1* and *Sphk2* expression profile in mouse eye tissues. (**B**) *S1pr1-3* expression profile in mouse eye tissues. (**C**) *Sphk1-2* expression profile in embryonic and adult whole mouse eyes. (**D**) *S1pr1-3* expression profile in embryonic and adult whole mouse eyes. (**E**) *Sphk1-2* expression profile in developing mouse retinas from postnatal day 1 (P1) to P30. (**F**) *S1pr1-3* expression profile in developing mouse retinas from P1 to P30. Cor, cornea. I-CB, iris/ciliary body. ON, optic nerve. EC, eye cup. Ret, retina. E15, 15 day embryo. E18, 18 day embryo. 2M, 2 months aged. 7M, 7 months aged. P1, P7, P15 and P30 are postnatal day 1, 7, 15 and 30, respectively.

Next, we determined the expression and distribution of *Sphk1* and *Sphk2* as well as the major S1P receptors *S1pr1*, *S1pr2* and *S1pr3* in various mouse eye tissues. RNA was prepared from cleanly dissected mouse cornea, lens, iris-ciliary body, optic nerve, eye cups and retina, as described previously [50,51,52]. Using qRT-PCR, we observed high expression of *Sphk2* throughout the cornea, iris/ciliary body, optic nerve, eye cup and retina while *Sphk1* expression was minimal in all ocular tissues and highest in the retina and optic nerve (Figure 2A), which reflects our previous findings in rat tissue. We observed high expression of *S1pr1* mRNA in mouse retinas, which is also in line with our prior findings, and noted high *S1pr1* expression in the iris/ciliary body, optic nerve and eye cups (Figure 2B). *S1pr1* is highly expressed in vascular tissues [21,53,54]. Thus, the expression observed may, to some extent, reflect the vascular component of each specific eye tissue. Compared to *S1pr1*, *S1pr2* expression was very low in the various eye tissues. Yet, we did detect some expression in the iris-ciliary body, optic nerve, eye cup and retina (Figure 2B). *S1pr3*, on the other hand, was ubiquitously expressed in all of the eye tissues examined and displayed very high expression in the optic nerve (Figure 2B).

We further determined the expression levels of sphingosine kinases and S1P receptors in whole eyes of mice during embryonic development (E15 and E18) and adulthood (at 2 and 7 months) by using qRT-PCR. As seen in our previous rat data, *Sphk2* was highly expressed throughout early development and adulthood while *Sphk1* expression remained relatively low at all stages (Figure 2C). *S1pr1* expression was approximately two-fold higher in eyes isolated from adult mice when compared to embryonic eyes (Figure 2D). In contrast, the levels of *S1pr2* mRNA were lower in the developed eyes than the embryonic eyes. In addition, *S1pr3* mRNA were relatively constant during embryonic development and in adulthood (Figure 2D). *S1pr3* was more highly expressed than *S1pr2* at all ages (Figure 2D).

In the developing mouse retina, both *Sphk1* and *Sphk2* expression appear to increase during early retinal development and peak at adulthood levels by postnatal day 30 (P30) (Figure 2E). Retinal *S1pr1* receptor expression gradually increases from P1 to P15 (Figure 2F), which could be related to retinal vascular development in this tissue. The retina is initially avascular when a mouse is born. However, starting at P1, the vasculature grows radially from the center of the retina towards the periphery until the retina reaches its mature size at around P15 [55,56]. *S1pr1* expression in the retina may, therefore, reflect the gradual increase of the vascular volume in the retina. *S1pr2* had lower levels of expression through all stages of retinal development while *S1pr3* expression was relatively high and constant at all time points (Figure 2F). To monitor stages of retinal development, we evaluated the expression of the markers *Elovl4* and *Rhodopsin* and found that they follow the expected pattern (Supplementary Figure S1).

### 2.3. Localization of Major S1P Receptors and Sphingosine Kinases in Rat Eye Tissues and Retina

#### 2.3.1. Localization of SPHK1 and SPHK2

**Figure 3:**
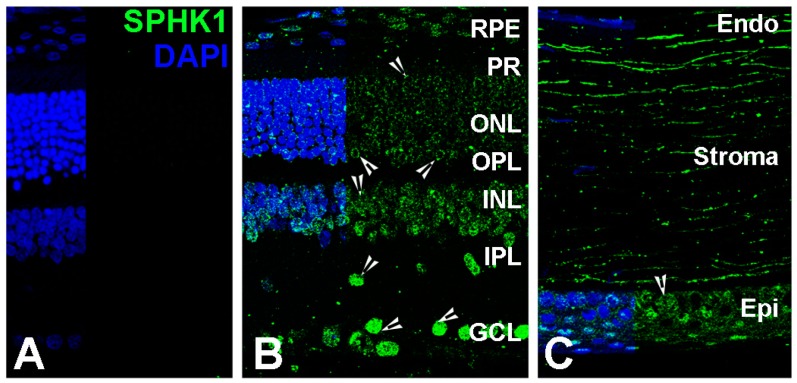

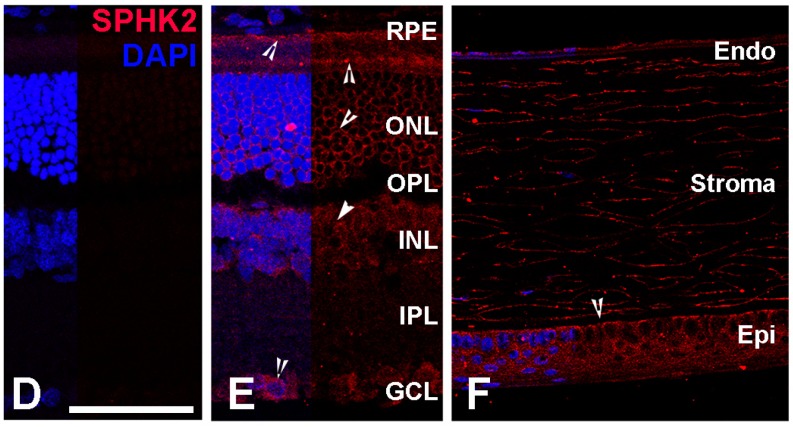
Localization of SPHK1 and SPHK2 in adult (2–7 months old) rat ocular tissues. SPHK1 and SPHK2 localization was detected by anti-SPHK1 (green) and anti-SPHK2 (red) antibodies. (**A**) Rat retinal section in which the anti-SPHK1 antibodies were replaced with normal rabbit serum. The layers were marked and served as a negative control. (**B**) Retinal section of SD rat eye and SPHK1 localization in different layers is shown by arrowheads. (**C**) Corneal section of SD rat eye and SPHK1 localization in different layers is shown by arrowheads. (**D**) Rat retinal section in which the anti-SPHK2 antibodies were replaced with normal rabbit serum. The layers were marked and served as a negative control. (**E**) Retinal section of SD rat eye and SPHK2 localization in different layers is shown by arrowheads. (**F**) Corneal section of SD rat eye and SPHK2 localization in different layers is shown by arrowheads. CB, ciliary body. RPE, retinal pigment epithelium. PR, photoreceptor. ONL, outer nuclear layer. OPL, outer plexiform layer. INL, inner nuclear layer. IPL, inner plexiform layer. GCL, ganglion cell layer. Endo, endothelium. Epi, epithelium. Scale = 50 µm.

To observe the localization of SPHK1 and SPHK2 in the retina and cornea, we performed immunohistochemistry (IHC) on sections of rat eyes taken through the vertical meridian and the optic nerve head using anti-SPHK1 and anti-SPHK2 antibodies. IHC localization of SPHK1 showed expression throughout the retina with strong staining in the ganglion cell bodies, large puncta in the IPL and extranuclear staining in photoreceptor cell bodies (Figure 3B—arrowheads). SPHK1 also showed expression in the corneal epithelium (Figure 3C—arrowheads). No-primary controls for corneal staining is shown in supplemental figures (Figure S2A). SPHK2 expression was observed throughout the retina notably in the RPE, ONL, INL and GCL (Figure 3E—arrowheads). We also noted some SPHK2 expression in the corneal epithelium (Figure 3F—arrowheads).

#### 2.3.2. Localization of S1PR1

**Figure 4:**
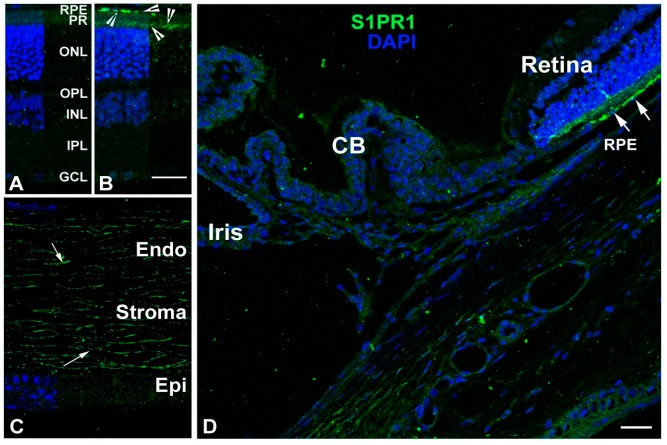
Localization of S1PR1 in adult (2–7 months old) rat ocular tissues. S1PR1 localization (green) was detected with anti-S1PR1 antibodies. (**A**) Rat retinal section in which the anti-S1PR1 antibodies were replaced with normal rabbit serum. The layers were marked and served as a negative control. (**B**) Retinal section of SD rat eye and S1PR1 localization in different layers is shown by arrowheads. (**C**) Corneal section of SD rat eye with S1PR1 labeling. (**D**) Partial anterior segment of eye containing ciliary epithelial cells, trabecular meshwork and Schlemm’s canal. S1PR1 localization is shown by arrowheads. RPE, retinal pigment epithelium. PR, photoreceptor. ONL, outer nuclear layer. OPL, outer plexiform layer. INL, inner nuclear layer. IPL, inner plexiform layer. GCL, ganglion cell layer. Endo, endothelium. Epi, epithelium. CB, ciliary body. Scale = 25 µm.

Little is known about the localization of S1PR1 in the neural retina and other eye tissues. We detected prominent labeling on retinal pigment epithelial (RPE) cells and photoreceptor cells with an anti-S1PR1 receptor antibody but not an IgG control (Figure 4A,B, arrowheads). However, we did not detect expression of S1PR1 in other parts of the eye such as the iris, lens (data not shown), or ciliary body (Figure 4C,D). In general, no localization of S1PR1 was noticed in the cornea. Corneal stromal labeling could be non-specific binding of the fluorescent antibody to the collagenous stroma (Figure 4C, arrows), as we did not detect this labeling in no-primary controls (Supplementary Figure S2B).

#### 2.3.3. Localization of S1PR2

**Figure 5:**
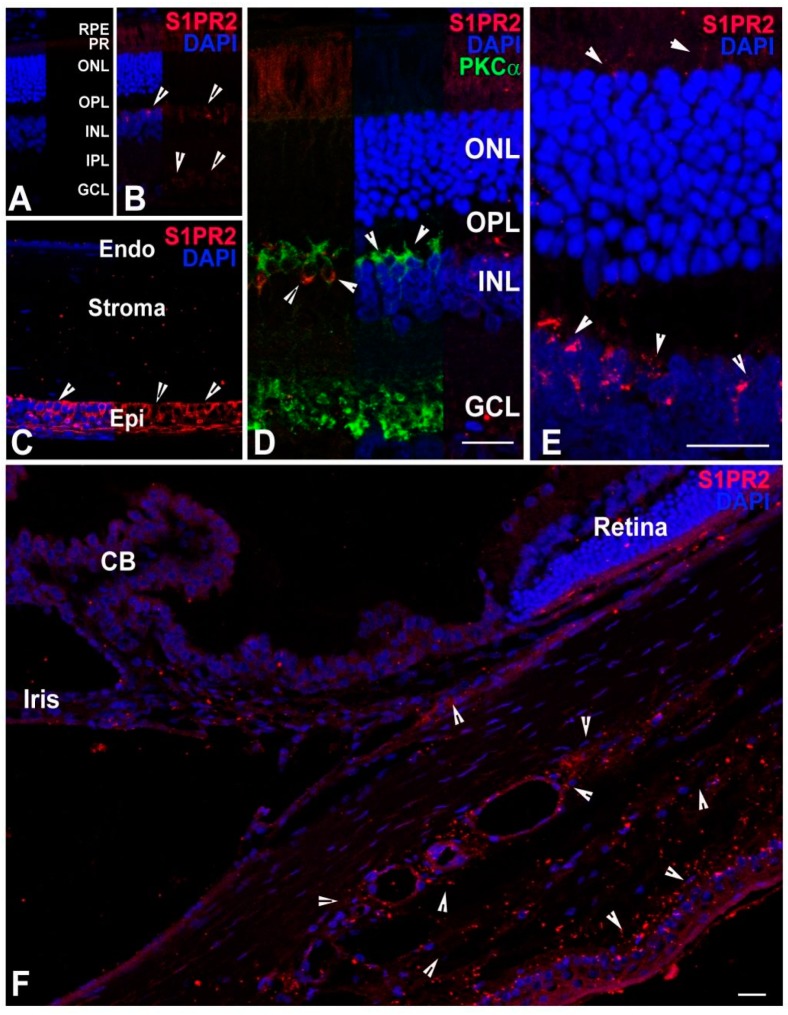
Localization of S1PR2 in adult (2–7 months old) rat ocular tissues. S1PR2 localization (red) was detected by anti-S1PR2 antibodies. (**A**) Rat retinal section in which the anti-S1PR2 antibodies were replaced with normal goat serum. The layers were marked and served as a negative control. (**B**) Retinal section of SD rat eye and S1PR2 localization in different layers is shown by arrowheads. (**C**) Corneal section of SD rat eye and S1PR2 localization in different layers is shown by arrowheads. (**D**) Magnified view of SD rat retinal section and S1PR2 localization with respect to PKCα in the INL and OPL layer, which is shown by arrowheads. (**E**) Magnified view of SD rat retinal section with emphasis on inner nuclear layer (INL) localization of S1PR2 shown by arrowheads. (**F**) Partial anterior segment of SD rat eye containing ciliary epithelial cells, Trabecular meshwork and Schlemm’s canal. S1PR2 localization is shown by arrowheads. CB, ciliary body. RPE, retinal pigment epithelium. PR, photoreceptor. ONL, outer nuclear layer. OPL, outer plexiform layer. INL, inner nuclear layer. IPL, inner plexiform layer. GCL, ganglion cell layer. Endo, endothelium. Epi, epithelium. Scale = 25 µm.

Using IHC, we found that an anti-S1PR2 antibody instead of IgG is labeled the very top layer of cells of the inner nuclear layer (INL) of rat retinal sections (Figure 5A,B,D,E). Since the INL consists primarily of bipolar cells, we sought to determine if the S1PR2 receptor colocalizes with PKCα, which is a marker of bipolar cell dendrites. We found that S1PR2 was present on the cell body of bipolar cells but did not overlap with the dendrites (Figure 5D, arrowheads). We also found that anti-S1PR2 antibodies labeled the corneal epithelium but not the corneal endothelium or stroma (Figure 5C). Lastly, punctate labeling of S1PR2 was prominent around the large vacuoles and Schlemm’s canal in the outflow pathway (Figure 5F, arrowheads).

#### 2.3.4. Localization of S1PR3

**Figure 6:**
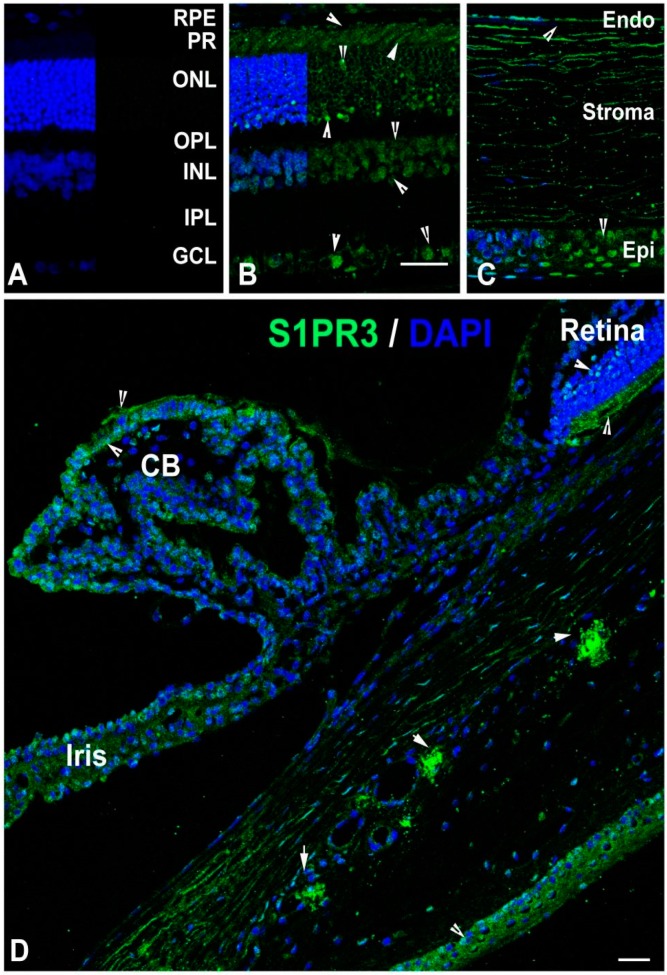
Localization of S1PR3 in adult (2–7 months old) rat ocular tissues. S1PR3 localization (green) was detected by anti-S1PR3 antibodies. (**A**) The rat retinal section in which the anti-S1PR3 antibodies were replaced with normal rabbit serum. The layers were marked and served as a negative control. (**B**) The retinal section of SD rat eye and S1PR3 localization in different layers is shown by arrowheads. (**C**) The corneal section of SD rat eye and S1PR3 localization in different layers is shown by arrowheads. (**D**) Partial anterior segment of SD rat eye containing ciliary epithelial cells, trabecular meshwork and Schlemm’s canal. The S1PR3 localization is shown by arrowheads. CB, ciliary body. RPE, retinal pigment epithelium. PR, photoreceptor. ONL, outer nuclear layer. OPL, outer plexiform layer. INL, inner nuclear layer. IPL, inner plexiform layer. GCL, ganglion cell layer. Endo, endothelium. Epi, epithelium. Scale = 25 µm.

Using IHC on SD rat retinal sections, we detected expression of S1PR3 in the ganglion cells and photoreceptor cells along with some expression in the photoreceptor inner segment region (inner nuclear layer) (Figure 6A,B, arrowheads). In the other parts of the eye, we observed strong anti-S1PR3 receptor staining of the corneal epithelium and some staining of the corneal endothelium (Figure 6C, arrowheads). Although the S1PR3 receptor was not detected in the corneal stroma (Figure 6C), it was observed in the pigmented epithelial cells of the ciliary body and iris (Figure 6D, arrowheads). Focal clustered labeling was observed around the Schlemm’s canal (Figure 6D, arrowheads).

### 2.4. Expression and Distribution of Sphingosine Kinases and S1P Receptors in Light Stressed Retinas

**Figure 7:**
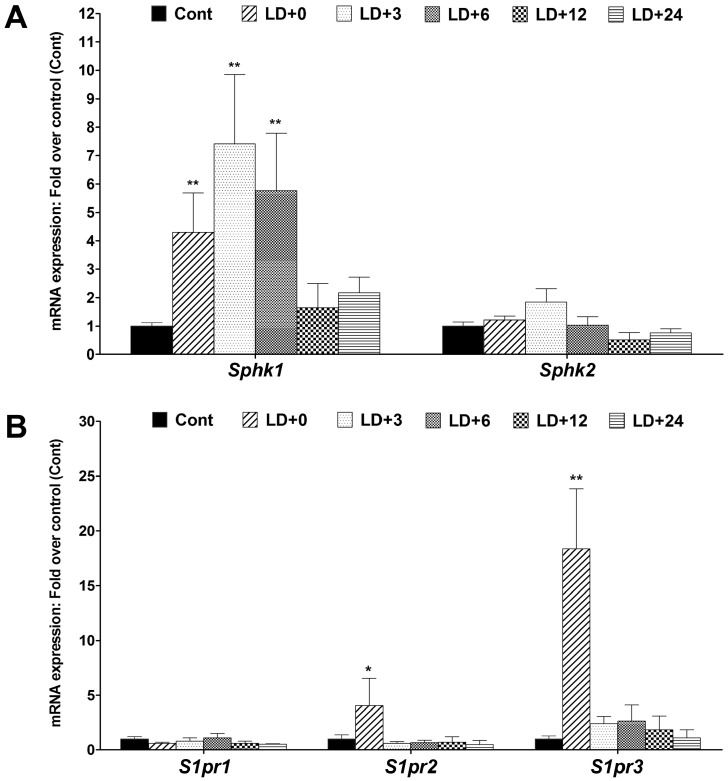
Light stress modulates sphingosine kinase (*Sphk*) 1 and 2 and sphingosine 1-phosphate receptor (*S1pr*) 1, 2 and 3 mRNA expression in adult (2–4 months old) rat retinas. Adult SD rats were exposed to damaging light at an intensity of 2700 lux for 6 h and their retinas were then harvested at different time points after light damage: 0 h (LD + 0), 3 h (LD + 3), 6 h (LD + 6), 12 h (LD + 12) and 24 h (LD + 24). Harvested samples were prepared for RNA extraction and subsequent qRT-PCR. (**A**) mRNA expression of *Sphk1* and *Sphk2* were presented as fold over control. (**B**) mRNA expression of *S1pr1*, *S1pr2* and *S1pr3* presented as fold over control. The expression data were calculated by dCt methods and compared to the expression of control housekeeping genes. The data presented are the mean of three independent experiments (±SE).

In order to examine the expression of *Sphk1-2* and the three major S1P receptors under light stress, we used our light-damaged rat retina model in which photoreceptor cell death occurs by apoptosis mainly due to oxidative stress induced by intense light exposure. Apoptosis starts after 8–12 h and by 24 h almost all of the photoreceptor cells in the central retina enter into apoptosis [57,58,59,60]. Using qRT-PCR, we observed a significant and sustained increase in *Sphk1* mRNA expression following light damage. Notably, *Sphk1* mRNA was elevated by four-fold, seven-fold and six-fold at LD + 0, LD + 3 and LD + 6, respectively, and began trending back toward baseline at 12 h (*p* < 0.01, Figure 7A). However, only a slight, non-significant elevation in *Sphk2* expression was recorded following light damage (Figure 7A). *S1pr2* and *S1pr3* mRNA expression increased significantly immediately after light damage (LD+0) by five-fold (*p* < 0.05) and twenty-fold (*p* < 0.01), respectively (Figure 7B). *S1pr2* and *S1pr3* expression returned to basal levels after 3 h and *S1pr1* expression remained constant throughout the experiment (Figure 7B).

**Figure 8:**
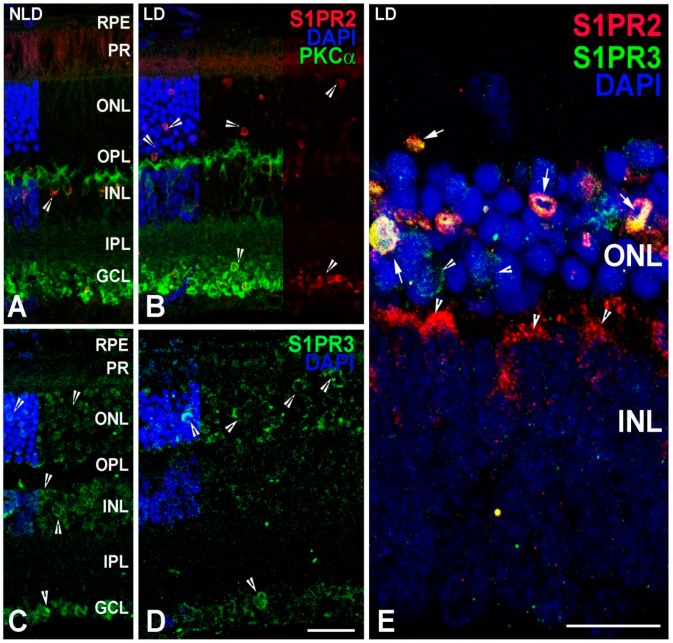
Localization of S1PR2, S1PR3 and PKCα in light damaged (LD) and non-light damaged (NLD) adult (2–4 months old) rat retinal tissues. (**A**) Retinal section of NLD SD rat eye with S1PR2 localization in different layers shown by arrowheads. (**B**) Retinal section of LD SD rat eye with S1PR2 localization in different layers shown by arrowheads. (**C**) Retinal section of NLD SD rat eye with S1PR3 localization in different layers shown by arrowheads. (**D**) Retinal section of LD SD rat eye with S1PR3 localization in different layers shown by arrowheads. (**E**) Magnified view of LD SD rat eye showing localization of S1PR2 and S1PR3 in the ONL and INL with arrowheads. RPE, retinal pigment epithelium. PR, photoreceptor. ONL, outer nuclear layer. OPL, outer plexiform layer. INL, inner nuclear layer. IPL, inner plexiform layer. GCL, ganglion cell layer. Scale = 25 µm.

We further examined protein localization in the retina immediately after light damage (LD + 0). No labeling of S1PR2 was detected in the photoreceptor nuclei (ONL) in the no-light-damaged retina (NLD, Figure 8A). However, at LD + 0, we detected abundant expression of S1PR2 in the photoreceptor cells, which may be undergoing apoptosis (Figure 8B). S1PR2 labeling increased in the ganglion cells after light damage as well (Figure 8B, arrowhead). S1PR3 labeling also increased and intensely co-localized with S1PR2 in photoreceptor cells after light damage (Figure 8D,E).

## 3. Discussion

G-protein-mediated signaling via S1P receptors has recently been shown to be involved in various cellular and biochemical processes including cell migration, inflammation, protection from apoptosis, etc. [5,11,13,21,43,61]. S1P receptors have been shown to function in neurogenesis, inflammation, neovascularization and tissue fibrosis, which are all associated with many blinding eye diseases. It is, therefore, of great interest and importance to understand the function of sphingolipid metabolism and signaling in the eye. However, very limited information is available on the expression and function of sphingosine kinases and S1P receptors in the mammalian retina and other ocular tissues. To fill this gap in knowledge, we determined the retinal expression levels of the sphingosine kinases (SPHK1 and SPHK2) and major S1P receptors (S1PR1, S1PR2, S1PR3 and S1PR5) relative to other rat tissues. We then evaluated the expression and localization of these kinases and the three major S1P receptors in specific ocular tissues from rats and mice and measured changes in their expression and localization following light-induced eye damage (LD).

S1P receptor expression in mammalian eye tissues has been reported previously and mouse *S1pr2* expression in the retina has also been reported in the context of neovascularization. S1PR1-3, 5 also had expression demonstrated in human RPE, conjunctival fibroblasts and corneal fibroblasts [46,54,62]. The trend of S1P receptor expression in eye tissue follows the general pattern of other tissues [63,64] such as *S1pr1* > *S1pr3* > *S1pr2*. The optic nerve has very high expression of *S1pr3* and also the highest relative *Sphk1* expression levels (Figure 2A,B), which could be of importance in terms of understanding the roles of S1P signaling in many forms of human optic nerve degeneration diseases and glaucoma.

The S1P receptor expression has been studied in detail across different cardiac tissues and reveals similar patterns for many we observed. In both cardiac myocytes and vascular endothelial cells, *S1pr1* has the highest expression, followed by *S1pr3*, and then followed by *S1pr2*. In cardiac fibroblasts, *S1pr3* has higher expression than *S1pr1* and, in aortic muscle smooth cells, *S1pr2* dominates *S1pr1* and *S1pr3* [63]. It should be noted that the expression pattern of S1P receptors changes during development and differentiation, which is shown by Figure 2D,F and leads to different combinations on cells and tissues [33,53,65,66]. This diversity in receptor expression and activation of divergent signaling pathways may explain how S1P signals have such pleiotropic responses. We focused here on sphingosine kinase and S1P receptor expression in eye tissues to help elucidate their roles in development, differentiation of ocular tissues, and pathogenesis of various eye diseases.

We observed high expression of *S1pr1* mRNA in mouse and rat retinas (Figure 1B and Figure 2B). As mentioned earlier, *S1pr1* is highly expressed in the vasculature [21,53,54]. Therefore, *S1pr1* mRNA expression might reflect the amount of vasculature present in that tissue. qRT-PCR revealed increasing *Sphk1, Sphk2 and S1pr1* expression with the age in mouse retinas (Figure 2E,F), which could be due to the development of vasculature and/or the development of the outer segments. Using IHC, we found that the neural cells of the mouse eye express negligible levels of S1PR1. However, prominent expression was detected in the RPE-choroid interface and the photoreceptor outer segment (Figure 4). Future studies using mouse models that do not develop outer segments could prove useful in determining the role that S1PR1 plays in the outer segments.

S1PR2, on the other hand, has expression in some inner retinal neurons, which could be rod bipolar cells since those cells can also be labeled with PKCα. This specifically labels the dendrites of rod bipolar cells. However, the PKCα and S1PR2 labels do not overlap, which indicates the presence of S1PR2 on the cell membrane of the bipolar cells but not on the dendrites (Figure 5D). This bipolar localization of S1PR2 suggests that this receptor may participate in some form of neurotransmission of bipolar cells through Gi, Gq, or G12/13 pathways [16,20,67]. S1PR2 was also found to localize to the photoreceptor inner segments, which is specifically very close to the outer nuclear layer (Figure 5E). Photoreceptors are primary cilia and the other related primary ciliary structure are the hair cells of the mammalian cochlea. Knocking out S1PR2 causes hair cell degeneration and deafness in mice [67,68]. It will be very interesting to study photoreceptor development and functioning in the *S1pr2* knock-out mice to help understand the role of S1PR2 photoreceptor development and function.

Exogenous S1P has been shown to decrease the outflow facility in the entire eyes of porcine, human and mouse models [69,70,71]. Using several S1P receptor specific agonists and antagonists, S1PR2 activation by exogenous S1P was found to cause an increase in outflow resistance and higher intraocular pressure [69,70,71]. This could be a potential target for developing therapeutics for regulating intraocular pressure. For the first time, we detected labeling of S1PR2 in the trabecular meshwork cells and higher on the endothelial cells close to the Schlemm’s canal (Figure 5F). We also detected intense, focal localization of S1PR3 in some of the proximal endothelial cells to the Schlemm’s canal (Figure 6D). S1P signaling is gaining importance with regards to their role in the human aqueous humor outflow pathway and glaucoma [69,70,71]. Determination of the presence of these receptors in the outflow pathway may bear significant potential to discover novel pathways and mechanisms for glaucoma development.

We noticed S1PR3 expression also localized to other retinal cells besides the photoreceptor cells (Figure 6B). In the photoreceptor cells, S1PR3 localizes to the cell body but not to the retinal outer segment (Figure 6D,G). *S1pr3* has higher expression in the retina and it increases significantly in retinal light-stress (Figure 1, Figure 2 and Figure 7). However, nothing is known on its role in retinal physiology and diseases. There is a potential for discovering novel pathways and mechanisms related to retinal development and function by studying S1PR3 in the retina.

In the light-stressed retina, we observed *Sphk1, S1pr2* and *S1pr3* expression increases significantly [39] (Figure 7). Light stress especially affects the photoreceptor cells [57,59,72,73,74,75,76]. An increase in the expression of a gene in this condition suggests its association with photoreceptor cell physiology. We found, in the light-stressed retina, that S1P receptors specifically and intensely localized to the pycnotic photoreceptor nuclei, which are entering into apoptosis (Figure 8E). This could be a cellular mechanism to up-regulate cytoprotective S1P signaling to counter the apoptosis, which might even be a feedback regulation of ceramide induced apoptosis of photoreceptor cells. S1P has previously been proposed as a key regulator in photoreceptor development as a stimulator of proliferation. Inhibiting S1P synthesis was shown to block mitogenic effects of glial-derived neurotrophic factor (GDNF) [77]. We have shown intense light-induced ceramide generation can induce apoptosis in photoreceptor cells [39]. We reported the S1P level also increases with light stress, which intensifies the expression of the photoreceptor specific S1P receptors and suggests a definitive signaling in the stressed photoreceptor cells. This could also be related to the G-protein coupled action of S1P receptors to affect channel functioning in the stressed photoreceptor cells. Our light damage model showing upregulation of SPHK2 and S1P receptors may implicate a signal for the immune response [78].

In conclusion, our results unveiled novel patterns of sphingosine kinase and S1P receptor expression and localization in the mammalian eye and retina. In line with previous literature and as discussed above, our results suggest that S1P receptor signaling is involved in numerous key processes in the eye ranging from light-stress and apoptotic responses to retinal and vascular development. The combination of the background high expression of *S1pr1*, high *S1pr1* and *S1pr3* expression in the developing retina and increased expression of *S1pr2* and *S1pr3* in the retina immediately after light-induced stress suggests that sphingolipid signaling plays a role in both vision and apoptosis. S1PR2′s presence in the outflow pathway follows logically from past studies on S1P receptor antagonist drugs and S1PR2 specific agonists and has great implications for understanding and treating conditions that include elevated IOP in glaucoma. S1PR2 is also likely involved in neurotransmission in the eye based on its localization to cell membranes in bipolar cells. *S1pr3′*s presence in the retina and upregulation during light damage pose interesting questions for the receptor’s poorly understood functions in the eye. These data allow future research to elaborate on not only mechanisms behind the receptors’ function in the respective tissues but also identifies potential markers and therapeutic targets for numerous ocular diseases.

## 4. Materials and Methods

### 4.1. Animal Care and Tissues

All procedures were performed, according to the ARVO Statement for the Use of Animals in Ophthalmic and Vision Research and the University of Oklahoma Health Sciences Center (OUHSC) Guidelines for Animals in Research. All protocols were reviewed and approved by the Institutional Animal Care and Use Committees of the OUHSC and the Dean A. McGee Eye Institute (DMEI) (IACUC approval #: 15-015-B, approval date: 24 February 2015; IACUC approval # 13-060-T, approval date 16 June 2015). All the tissues used in this study were from albino Sprague Dawley (SD) rats, C57Bl6J mice and bovine retina. Mouse and rat retinal tissues used in this study for gene expression were harvested after overnight dark adaptation.

To study tissue distribution of S1P receptor expression, we prepared RNA from adult (2–7 months old) rat tissue. For every tissue harvest, the rats were first euthanized by carbon dioxide asphyxiation. The harvested forebrain, hindbrain, liver, heart, lungs, skin, testes, ovary, spleen and kidney was then snap frozen in liquid N_2_. Light-adapted and dark-adapted rat retinas were harvested under room-light and red-light, respectively, snap-frozen, and used for preparing rod outer segments (ROS). The whole eyes were harvested and fixed in Prefer fixative (Anatech Ltd., Battle Creek, MI, USA) for 20 to 40 min and embedded in paraffin. Additionally, 5 µm-thick sections were prepared from the fixed rat eyes and used for immunohistochemistry.

To study the distribution of gene expression in eye tissues, six to eight eyeballs were obtained from adult mice (2–3 months old) after euthanization and the distal-most 3-mm optic nerve cut from the scleral surface of the eyeballs (ON), posterior segment (PS), retina, iris-ciliary body (I-CB), lens and corneal tissues were collected after dissection. Eyeballs (6 to 8) from embryonic (E15 and E18) and adult (2 and 7 months) mice were collected to study the expression profile of S1P receptors in the whole eye during embryonic and adult stages. To study retinal expression during development, we collected 8 to 10 retinas from four to five C57Bl6J mice at postnatal days P1, P7, P15 and P30.

### 4.2. Light Damage of Albino Rat Retina

Adult (2–4 months old) SD rats were light damaged by being subjected to 2700 lux of white light for 6 h in specially fabricated light boxes, which was described in previous publications [39,59,60]. The retinas were harvested at various time points following the light exposure (0 h to 24 h). Eyes were also harvested at various time points, fixed in Prefer fixative (Anatech Ltd., Battle Creek, MI, USA) for 20 to 40 min, embedded in paraffin, and sectioned for conducting immunohistochemistry.

### 4.3. Gene Expression Analysis by Quantitative RT-PCR (qRT-PCR)

The RNA was isolated and purified from frozen rat tissues using PureLink^®^ Micro-to Midi Total RNA Purification System from Invitrogen (Carlsbad, CA, USA) and following the manufacturer’s protocol. Equal quantities (1.0 µg) of total RNA from each tissue were converted to first-strand cDNA using SuperScript III First-Strand Synthesis SuperMix (Invitrogen, Carlsbad, CA, USA) for RT-PCR. First-strand cDNA was used for qRT-PCR. Primers for qRT-PCR were intron-spanning (Supplemental Table S1). Quantitative PCR and melt-curve analyses were performed using iQ SYBR Green Supermix (Bio-Rad, Hercules, CA, USA) and an iCycler machine. Relative quantities of expression of the genes of interest in different samples were calculated by the comparative Ct (threshold cycle) value method [39,79,80].

### 4.4. Immunohistochemistry

Immunolabeling on paraffin sections was performed as described previously [39,51,52,80]. The sections were deparaffinized, rehydrated in PBS (phosphate-buffered saline), and nonspecific labeling was blocked using 10% normal horse serum in PBS for 2 h. Excess blocker was removed and the primary antibody was applied overnight at 4 °C. IHC antibodies were used as follows: SphK1 rabbit polyclonal [1:100–1:200] (Santa Cruz Biotechnology, Santa Cruz, CA, USA), Sphk2 goat polyclonal [1:100–1:200] (Santa Cruz Biotechnology, Santa Cruz, CA), S1PR1 rabbit polyclonal [1:200] (Cayman, Ann Arbor, MI, USA), S1PR2 goat polyclonal [1:100] (Santa Cruz Biotechnology, Santa Cruz, CA, USA), S1PR3 rabbit polyclonal [1:200] (Santa Cruz Biotechnology, Santa Cruz, CA, USA), PKCα rabbit polyclonal [1:200] (Cell Signaling, Danvers, MA, USA), and Rhodopsin mouse monoclonal [1:200] (Abcam, Cambridge, MA, USA). Sections were rinsed and incubated with appropriate secondary antibodies conjugated to fluorescence dye (Alexa flour, Invitrogen, Carlsbad, CA, USA) @ 1:1000 dilution for 1 h at room temperature to visualize labeling. Sections were rinsed and cover-slipped with ProLong Gold antifade reagent with DAPI (Invitrogen, Carlsbad, CA, USA). Confocal microscopy was performed using an Olympus FluoView FV500 confocal microscopy system (Olympus Microsystems, Center Valley, PA, USA). To ensure quantitative image quality, laser power, pinhole settings, photomultiplier tube settings, and intensity thresholds were kept constant for the used antibody.

### 4.5. Statistical Analyses

Statistical analyses were performed using GraphPad Prism 5.0 software (GraphPad Software, Inc.; La Jolla, CA, USA). The quantitative data are expressed as mean ± SE for each group. One-way or two-way ANOVA and Student’s and paired t-tests were performed to assess differences between means.

## Supplementary Materials

Supplementary materials can be found at http://www.mdpi.com/1422-0067/19/12/3885/s1.

## Abbreviations

S1P	Sphingosine 1-Phosphate
S1PR	Sphingosine 1-Phosphate Receptor
SPHK	Sphingosine Kinase
Cer	Ceramide
qRT-PCR	Quantitative Reverse Transcriptase Polymerase Chain Reaction
RPE	Retinal Pigment Epithelium
IHC	Immunohistochemistry
PR	Photoreceptor
ONL	Outer Nuclear Layer
OPL	Outer Plexiform Layer
INL	Inner Nuclear Layer
IPL	Inner Plexiform Layer
GCL	Ganglion Cell Layer
Endo	Endothelium
Epi	Epithelium
CB	Ciliary Body
