# Supplementary material for: Characterizing Sphingosine Kinases and Sphingosine 1-Phosphate Receptors in the Mammalian Eye and Retina
Source: Int J Mol Sci. 2018 Dec 5;19(12):3885. doi: 10.3390/ijms19123885 (PMC6321283; doi:10.3390/ijms19123885)
Supplement: Supplementary file 1 [file ijms-19-03885-s001.pdf]

Supplemental Information

Supplemental Table 1. Table of Forward and Reverse Primers for qRT-PCR. Degenerate primers that recognize both mouse and rat genes were designed and used for the qRT-PCR experiments.

Gene	Forward Primer (5'-3')	Reverse Primer (5'-3')
Sphk1	GATGCATGAGGTGGTGAATG	AACAGCAGTGTGCAGTTGAT
Sphk2	GAAGGCATTGTCACTGTGTC	GCAGAGAAGAAGCGAGCAGT
S1pr1	GGATCGCGCGGTGTAGAC	GCTAGAGGGCGAGGTTGAG
S1pr2	CACTATGTGCTCTGCGTGGT	GGCGATGTAGGCATATGCA
S1pr3	CCTCATCACCACCATCCTCT	TGGAGTAGAGGGGCAAGATG
S1pr5	CCAAGGCCTATGTGCTCTTC	GTTGGAGGAGTCTTGGTTGC
Rhodopsin	CTTCCTGATCTGCTGGCTTC	ACAGTCTCTGGCCAGGCTTA
Elovl4	TCCAGAAATATCTTTGGTGG	GTTAAGGCCCAGTTCAATT
Rpl19	TCACAGCCTGTACCTGAAGG	TCGTGCTTCCTTGGTCTTAG

Supplement Figure 1

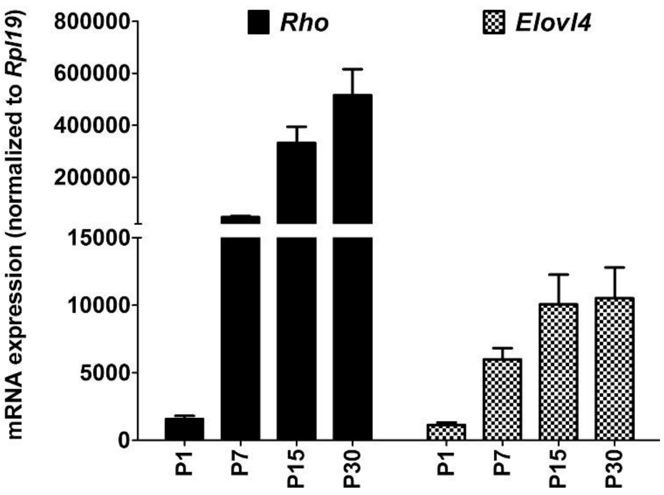

Figure S1. The expression of Rhodopsin (*Rho*) and Elongation of very long chain fatty acids 4 (*Elovl4*) in developing mouse retinas. Rhodopsin express only in the rod photoreceptor and Elovl4 in both rod and cone photoreceptors; both are localized to the outer segments of the photoreceptors. Their expression increases starting at P7, a time point at which photoreceptor cells start developing outer segments. Expression increases until the photoreceptors mature at P30. Expression of Rho and Elovl4 were used here as markers for developing mouse retinas. For each time point and gene, the mean of three independent qRT-PCR experiments (± SE) is shown, normalized to the expression of the ribosomal protein Rpl19. P1, P7, P15 and P30 are postnatal day 1, 7, 15, and 30, respectively.

Supplement Figure 2

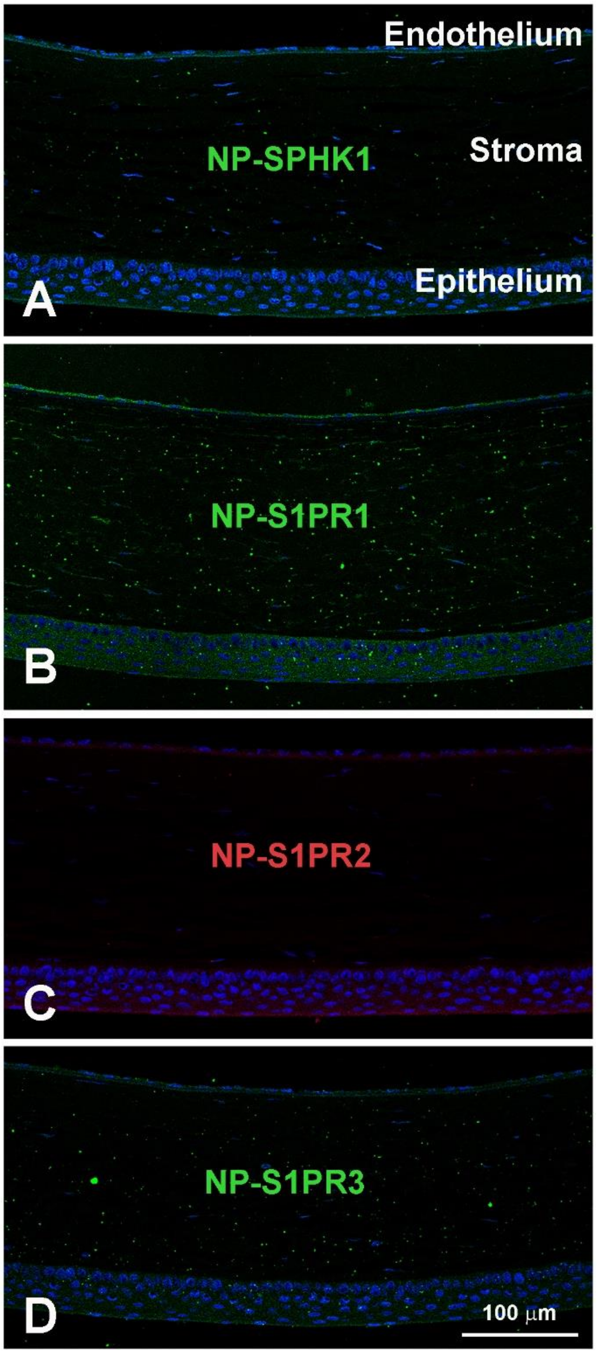

Figure S2. Corneal sections of SD rat eyes were treated with DAPI and immunofluorescent secondary antibodies with no primary antibody treatment (NP) in order to visualize nonspecific binding in the cornea.
